# Supplementary material for: Colorectal cancer liver metastatic growth depends on PAD4-driven citrullination of the extracellular matrix
Source: Nat Commun. 2018 Nov 14;9:4783. doi: 10.1038/s41467-018-07306-7 (PMC6235861; doi:10.1038/s41467-018-07306-7)
Supplement: Supplementary file 1 — Supplementary Information [file 41467_2018_7306_MOESM1_ESM.pdf]

Supplementary Information

to

Colorectal cancer liver metastatic growth depends on PAD4-driven citrullination of the extracellular matrix

by Yuzhalin et al.

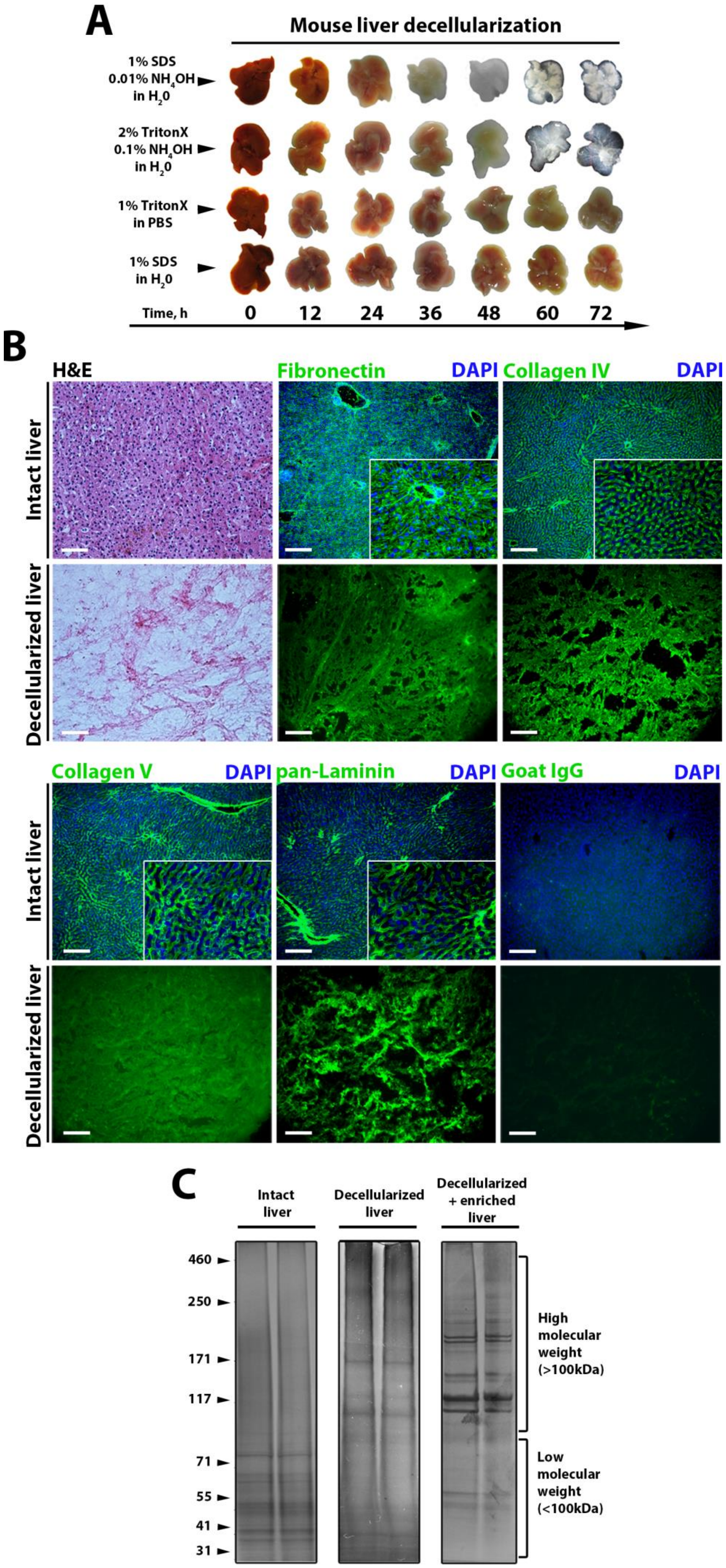
A
Mouse liver decellularization
1% SDS 0.01% NH4OH in H20
2% TritonX 0.1% NH4OH in H20
1% TritonX in PBS
1% SDS in H20
Time, h
0
12
24
36
48
60
72
B
H&E
Fibronectin
DAPI
Collagen IV
DAPI
Intact liver
Decellularized liver
Collagen V
DAPI
pan-Laminin
DAPI
Goat IgG
DAPI
Intact liver
Decellularized liver
C
Intact liver
Decellularized liver
Decellularized + enriched liver
460
250
171
117
71
55
41
31
High molecular weight (>100kDa)
Low molecular weight (<100kDa)

Supplementary Figure 1. Optimization of ECM isolation. **A.** Representative images of mouse livers decellularized by the indicated solutions after indicated times **B**. Intact or decellularized mouse livers were stained with H&E or by immunohistochemistry for the indicated ECM proteins. Isotype control staining was performed using goat IgG. Scale bar = 100 µm. **C**. Total protein extracted from intact (left), decellularized (middle), and decellularized and biochemically enriched (right) mouse livers was resolved by SDS-PAGE and silver stained. High and low molecular weight areas were considered >100 kDa and <100 kDa, respectively. Experiments were performed in parallel in duplicate. H – hours. H&E – hematoxylin and eosin, PBS – phosphate-buffered saline.

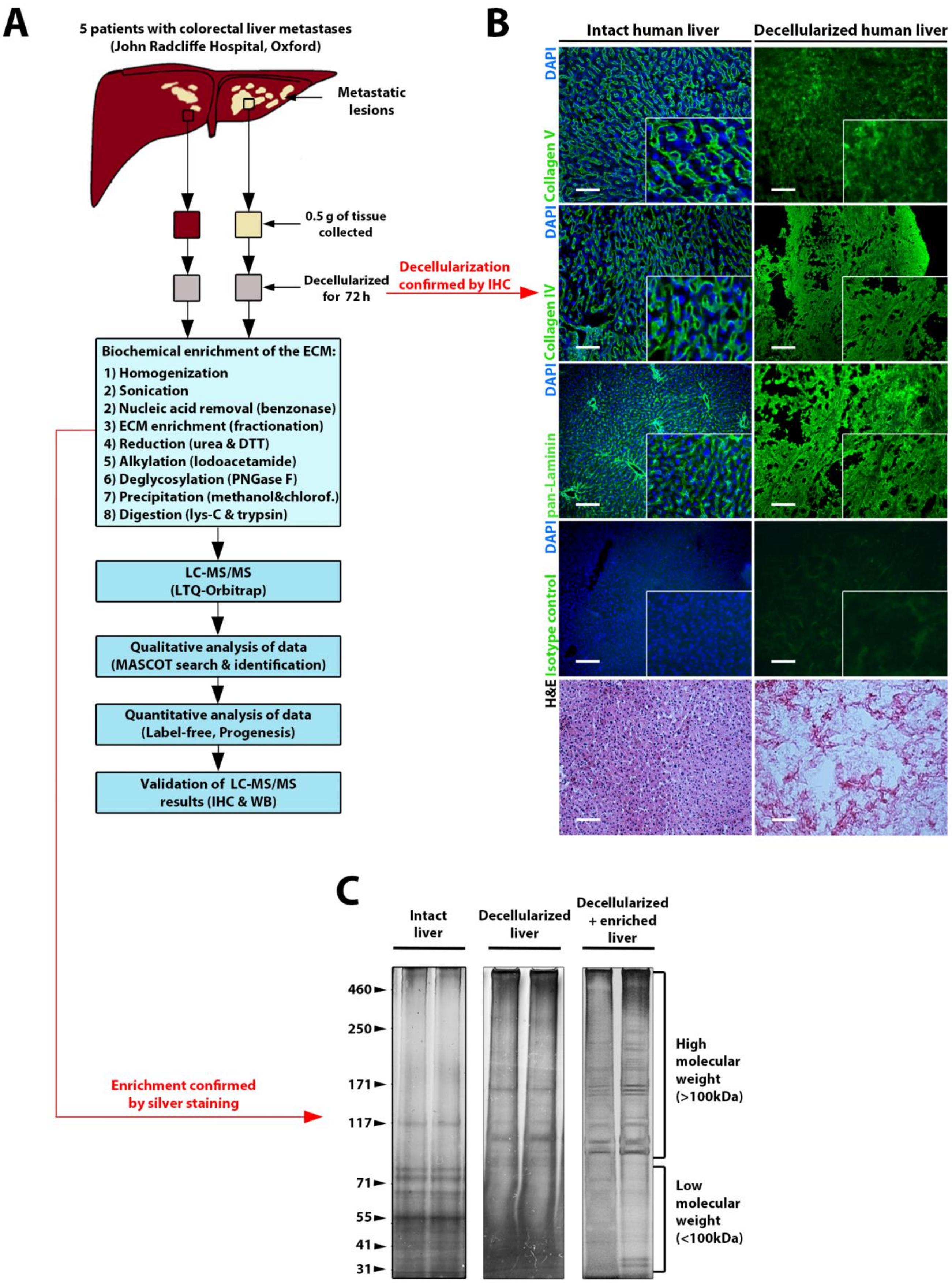

Supplementary Figure 2. Study design and preparation of human ECM-enriched decellularized matrices of CRC hepatic metastases and adjacent unaffected livers. **A**. Study pipeline describing ECM enrichment protocol and proteomics analysis. **B**. Human normal liver tissues, decellularized or intact, were stained for the indicated proteins, isotype control antibody, or with H&E. Scale bar = 100 µm. **C**. Total protein extracted from intact (left), decellularized (middle), and decellularized and biochemically enriched (right) human liver tissue adjacent to metastasis was resolved by SDS-PAGE and silver stained. High and low molecular weight areas were considered >100 kDa and <100 kDa, respectively. Experiments were performed in parallel in duplicate.

IHC – immunohistochemistry, WB – western blotting. LC-MS/MS – liquid chromatography–mass spectrometry, DTT – dithiothreitol. H&E – hematoxylin and eosin, h – hours.

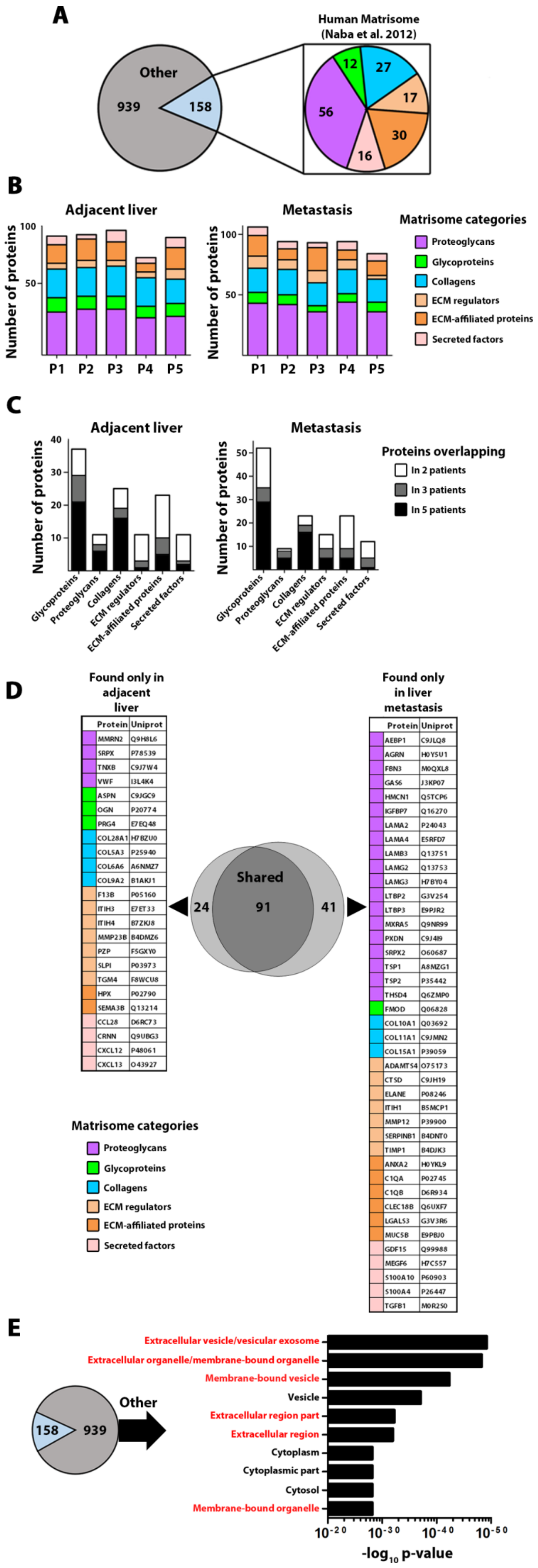
A
Human Matrisome
(Naba et al. 2012)
Other
939
158
12
27
17
56
30
16
B
Adjacent liver
Metastasis
Number of proteins
100
50
P1 P2 P3 P4 P5
Matrisome categories
Proteoglycans
Glycoproteins
Collagens
ECM regulators
ECM-affiliated proteins
Secreted factors
C
Adjacent liver
Metastasis
Number of proteins
40
30
20
10
50
Glycoproteins
Proteoglycans
Collagens
ECM regulators
ECM-affiliated proteins
Secreted factors
Proteins overlapping
In 2 patients
In 3 patients
In 5 patients
D
Found only in adjacent liver
Protein Uniprot
MMRN2 Q9H8L6
SRPX P78539
TNXB C9J7W4
VWF I3L4K4
ASPN C9JGC9
OGN P20774
PRG4 E7EQ48
COL28A1 H7BZU0
COL5A3 P25940
COL6A6 A6NMZ7
COL9A2 B1AKJ1
F13B P05160
ITIH3 E7ET33
ITIH4 B7ZKJ8
MMP23B B4DMZ6
PZP F5GXY0
SLPI P03973
TGM4 F8WCU8
HPX P02790
SEMA3B Q13214
CCL28 D6RC73
CRNN Q9UBG3
CXCL12 P48061
CXCL13 O43927
Shared
24
91
41
Found only in liver metastasis
Protein Uniprot
AEBP1 C9JLQ8
AGRN H0Y5U1
FBN3 M0QXL8
GAS6 J3KP07
HMCN1 Q5TCP6
IGFBP7 Q16270
LAMA2 P24043
LAMA4 E5RFD7
LAMB3 Q13751
LAMG2 Q13753
LAMG3 H7BY04
LTBP2 G3V254
LTBP3 E9PJR2
MXRA5 Q9NR99
PXDN C9J4I9
SRPX2 O60687
TSP1 A8MZG1
TSP2 P35442
THSD4 Q6ZMP0
FMOD Q06828
COL10A1 Q03692
COL11A1 C9JMN2
COL15A1 P39059
ADAMTS4 O75173
CTSD C9JH19
ELANE P08246
ITIH1 B5MCP1
MMP12 P39900
SERPINB1 B4DNT0
TIMP1 B4DJK3
ANXA2 H0YKL9
C1QA P02745
C1QB D6R934
CLEC18B Q6UXF7
LGALS3 G3V3R6
MUC5B E9PBJ0
GDF15 Q99988
MEGF6 H7C557
S100A10 P60903
S100A4 P26447
TGFB1 M0R2S0
Matrisome categories
Proteoglycans
Glycoproteins
Collagens
ECM regulators
ECM-affiliated proteins
Secreted factors
E
Other
158
939
Extracellular vesicle/vesicular exosome
Extracellular organelle/membrane-bound organelle
Membrane-bound vesicle
Vesicle
Extracellular region part
Extracellular region
Cytoplasm
Cytoplasmic part
Cytosol
Membrane-bound organelle
10-20 10-30 10-40 10-50
-log10 p-value

Supplementary Figure 3. Characterization of the matrisome of human CRC hepatic metastases by qualitative proteomics. **A**. Decellularized and ECM-enriched fractions obtained from human CRC hepatic metastases and paired adjacent unaffected liver tissues (*n*=5 per group) were analyzed by LC-MS/MS. Left pie chart displays the total number of proteins identified by MASCOT search in at least 1 individual sample and by at least 2 peptides. Right pie chart displays proteins identified as the ECM in accordance with categorization by Naba et al.[13] **B**. Bar graphs show inter-sample proteomics variability between individual samples in metastasis and adjacent liver groups within each ECM category. **C**. Bar graphs show the overlap of identified proteins in individual patients across ECM categories **D.** Lists of matrisome proteins identified exclusively in metastasis (right) or adjacent liver groups (left) in at least 1 patient. **E**. Proteins categorized as non-ECM proteins were processed through Panther Gene Ontology (GO) enrichment analysis. Top 10 enriched GO categories are shown. ECM-related GO categories are highlighted in red. P – patient, ECM – extracellular matrix.

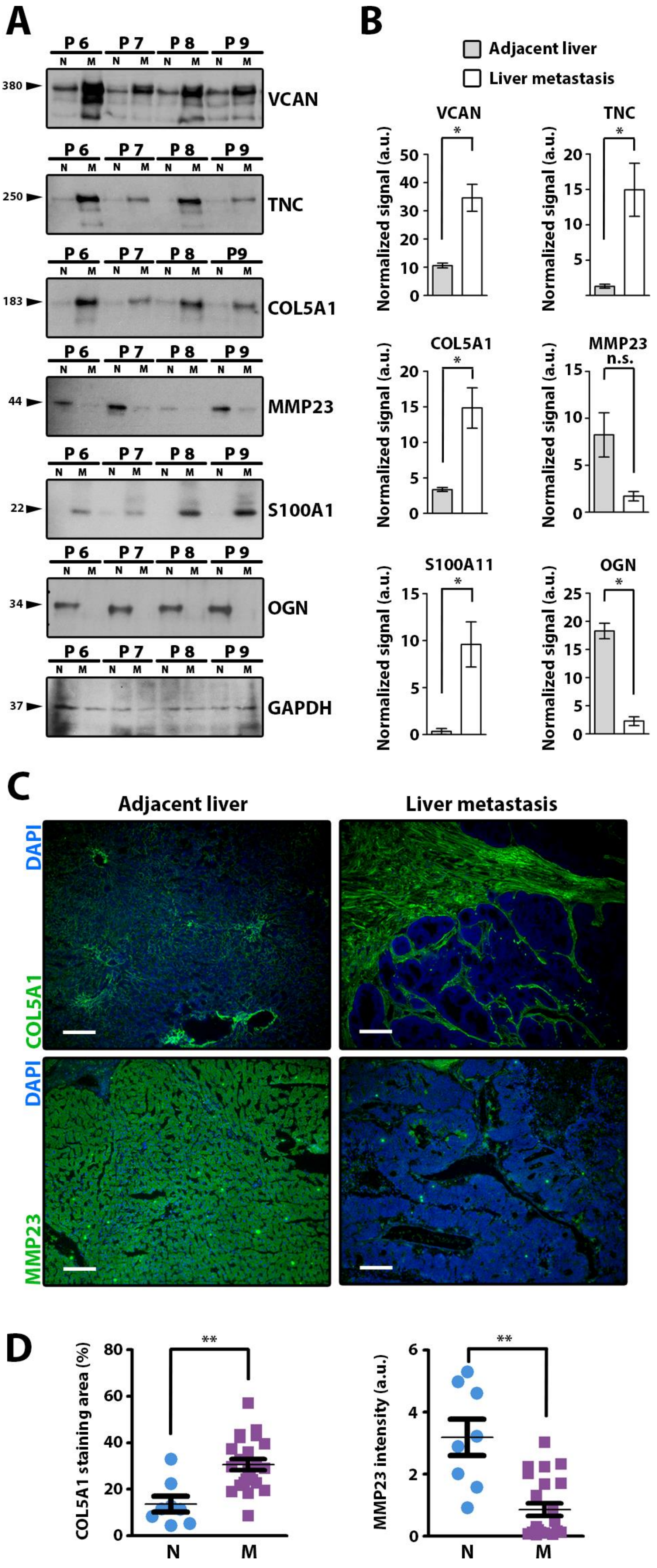

A
P 6
P 7
P 8
P 9
N
M
380
VCAN
250
TNC
183
COL5A1
44
MMP23
22
S100A1
34
OGN
37
GAPDH
B
Adjacent liver
Liver metastasis
VCAN
TNC
COL5A1
MMP23
n.s.
S100A11
OGN
Normalized signal (a.u.)
C
Adjacent liver
Liver metastasis
DAPI
COL5A1
MMP23
D
COL5A1 staining area (%)
MMP23 intensity (a.u.)
**

Supplementary Figure 4. Validation of proteomics results. **A.** Tissue lysates extracted from a different set of human CRC hepatic metastases and paired adjacent unaffected liver tissues (*n*=4 per group) were immunoblotted for the indicated ECM proteins. GAPDH was used as a loading control. **B**. Densitometry analysis of protein bands detected in **A.** Densitometry was normalized to GAPDH. **C.** Representative images of COL5A1 and MMP-23 staining of 23 CRC liver metastasis tissues (right) and 8 non-matched adjacent unaffected liver tissues (left). Scale bar = 100 µm. **D**. Quantification of COL5A1 staining area and MMP-23 intensity from the experiment in **C**. For **B**, error bars indicate s.e.m. (**P* < 0.05, n.s. = non-significant, Wilcoxon signed rank test). For **D**, error bars indicate s.e.m. (***P* < 0.01, Mann-Whitney U test). P – patient. N – adjacent unaffected liver, M – metastasis.

A **Identification of citrullinated peptides using PEAKS® software:**

```
R.C(+57.02)D(-18.01)R(+.98)NLVWNAGALHYSDEVEIIQGLTR.M
R.NFTAADWGQSR(+.98)DAEEAISQTIDTIVDMIK.N
K.GYR(+.98)GPE(+57.02)GPQ(+.98)GPPGHQGPPGPDEC(+57.02)EILDIIMK.M
K.GYR(+.98)GPE(+57.02)GPQGPPGHQ(+.98)GPPGPDEC(+57.02)EILDIIMK.M
K.G(+57.02)YR(+.98)GPEGPQ(+.98)GPPGHQGPPGPDEC(+57.02)EILDIIMK.M
```

COL6A1 peptides (Patient 5, metastasis)

R(+.98) = **Citrullinated arginine residue**

B

Citrullinated in adjacent liver only

Category	Protein	Uniprot	Citrullinated peptides: Adjacent liver	Citrullinated peptides: Metastasis
Proteoglycans	HSPG2	P98160	7	0
Collagens	COL6A2	P12110	3	0
ECM regulators	TGM2	P21980	2	0
Glycoproteins	ELN	P15502	1	0
Glycoproteins	LAMB2	P55268	1	0
Collagens	COL5A1	P20908	1	0
Collagens	COL22A1	Q8NFW1	1	0
Collagens	COL6A6	A6NMZ7	1	0
ECM affiliated proteins	KRT6A	P02538	1	0
ECM regulators	MTHFD1	P11586	1	0
Secreted factors	ZNF469	Q96JG9	1	0
Other	DSEL	Q8IZU8	1	0
Other	TUFM	P49411	1	0
Other	SYNCRIP	O60506	1	0
Other	HNRNPA2B1	P22626	1	0
Other	IGHA1	P01876	1	0
Other	PCBP4	P57723	1	0
Other	FRYL	O94915	1	0
Other	SEC24D	O94855	1	0
Other	ALB	P02768	1	0
Other	MYO1A	Q9UBC5	1	0

Citrullinated in both

Category	Gene name	Uniprot	Citrullinated peptides: Adjacent liver	Citrullinated peptides: Metastasis
Glycoproteins	FBN1	P35555	8	23
Glycoproteins	EMILIN1	Q9Y6C2	4	11
Collagens	COL1A1	P02452	6	10
Collagens	COL6A1	P12109	7	9
Glycoproteins	FN1	P02751	4	8
Collagens	COL1A2	P08123	6	6
Collagens	COL4A2	P08572	2	5
Collagens	COL4A1	P02462	1	5
Collagens	COL2A1	P02458	6	4
Other	VTN	P04004	3	3
Collagens	COL5A2	P05997	2	3
Glycoproteins	FGA	P02671	1	3
Collagens	COL6A3	P12111	4	2
Glycoproteins	FGB	P02675	3	2
Glycoproteins	TNC	P24821	3	2
Other	FUS	P35637	3	2
Collagens	COL3A1	P02461	2	2
Glycoproteins	LAMG1	P11047	1	2
Other	EEF1A1	P68104	1	2
Glycoproteins	FGG	P02679	1	1
Glycoproteins	TGFBI	Q15582	1	1

Citrullinated in metastases only

Category	Gene name	Uniprot	Citrullinated peptides: Adjacent liver	Citrullinated peptides: Metastasis
Other	ACTB	P60709	0	5
Other	ACTA1	P68133	0	3
Other	PFN1	P07737	0	3
Collagens	COL8A1	P27658	0	2
Collagens	COL11A1	P12107	0	2
Other	REV3L	O60673	0	2
Other	HNRNPU	Q00839	0	2
Glycoproteins	EFEMP1	Q12805	0	1
Glycoproteins	EMILIN2	Q9BXX0	0	1
Glycoproteins	FBLN5	Q9UBX5	0	1
Glycoproteins	LTBP2	Q14767	0	1
Glycoproteins	NID2	Q14112	0	1
ECM regulators	ELANE	P08246	0	1
ECM affiliated proteins	MUC2	Q02817	0	1
ECM affiliated proteins	KRT7	P08729	0	1
Other	PFKL	P17858	0	1
Other	DBNL	Q9UJU6	0	1
Other	HSPB1	P04792	0	1
Other	HSP90AA1	P07900	0	1
Other	OLFM4	Q6UX06	0	1
Other	PABPC3	Q9H361	0	1
Other	HSPA9	P38646	0	1
Other	HADHA	P40939	0	1
Other	TUBA1A	Q71U36	0	1

Glycoproteins; Proteoglycans; Collagens; ECM regulators; ECM affiliated proteins; Secreted factors; Other

Supplementary Figure 5. Identification of ECM-associated citrullinome of metastasis-bearing livers. **A.** PEAKS® software was utilized to quantify citrullinated peptides in the ECM of CRC hepatic metastases. Citrullinated arginine residues are designated as R(+.98) and marked in red. For illustration, shown are the citrullinated peptides of collagen 6 in the metastasis sample of patient 5. **B.** Tables display the ECM-associated citrullinome of metastasis-bearing livers. Citrullinated peptides were assigned to their corresponding proteins and ranked in accordance with ECM categorization and their abundance in metastasis and adjacent liver groups. Shown are proteins identified as exclusively citrullinated in metastasis (right), or adjacent liver (left), or found citrullinated in both groups (middle). ECM – extracellular matrix.

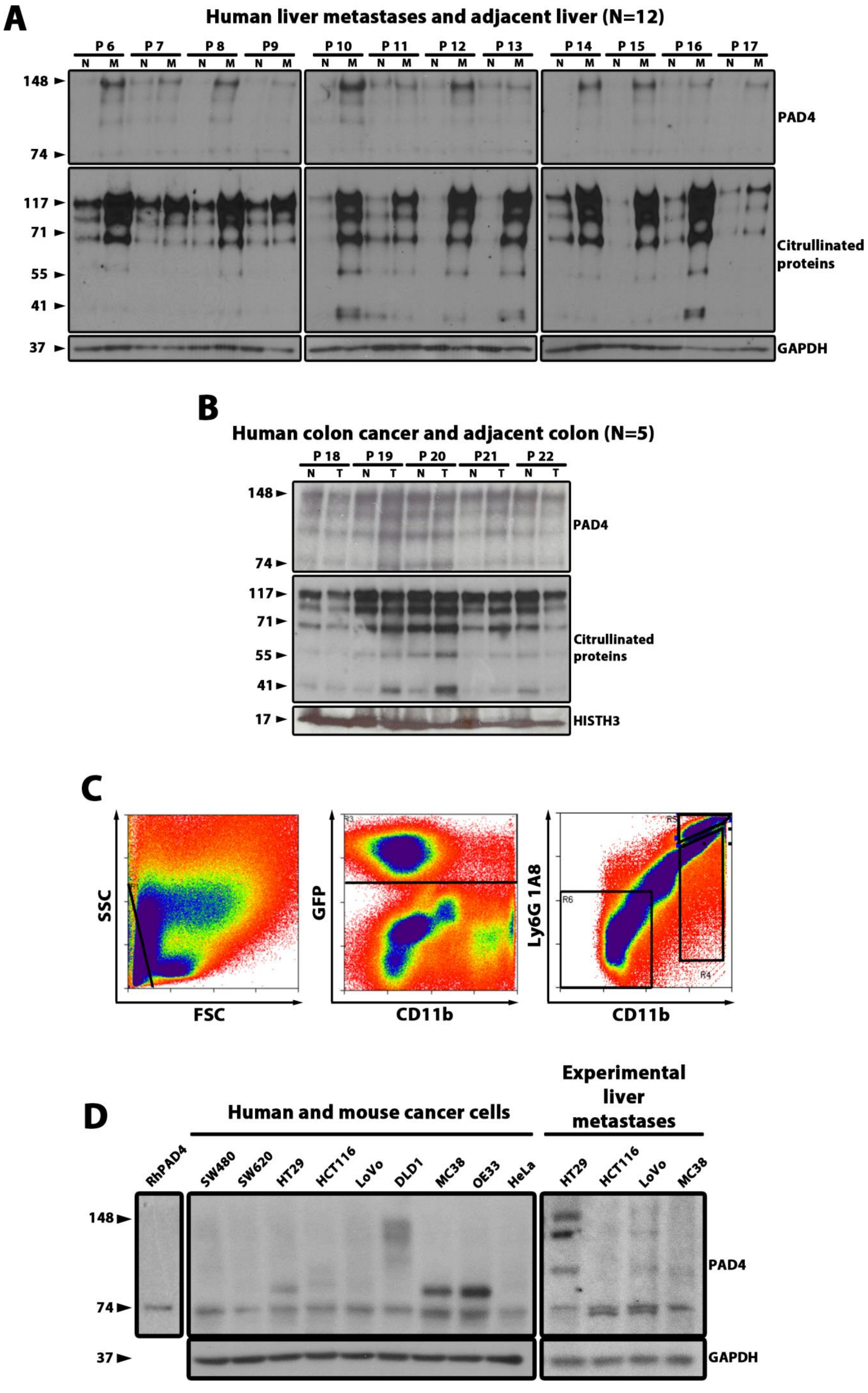
A
Human liver metastases and adjacent liver (N=12)
P 6
P 7
P 8
P9
P 10
P 11
P 12
P 13
P 14
P 15
P 16
P 17
N M
148 ►
74 ►
PAD4
117 ►
71 ►
55 ►
41 ►
Citrullinated proteins
37 ►
GAPDH
B
Human colon cancer and adjacent colon (N=5)
P 18
P 19
P 20
P21
P 22
N T
148 ►
74 ►
PAD4
117 ►
71 ►
55 ►
41 ►
Citrullinated proteins
17 ►
HISTH3
C
SSC
FSC
GFP
CD11b
Ly6G 1A8
CD11b
R3
R5
R6
R4
D
Human and mouse cancer cells
Experimental liver metastases
RhPAD4
SW480
SW620
HT29
HCT116
LoVo
DLD1
MC38
OE33
HeLa
HT29
HCT116
LoVo
MC38
148 ►
74 ►
PAD4
37 ►
GAPDH

Supplementary Figure 6. Characterization of PAD4 expression and citrullination prevalence in human liver metastases and primary CRC. **A.** Immunoblotting for PAD4 and citrullinated proteins performed on tissue lysates extracted from hepatic metastasis tissues and adjacent unaffected liver tissues (*n*=12 per group). GAPDH was used as a loading control. **B**. Immunoblotting for PAD4 and citrullinated proteins performed on tissue lysates extracted from primary CRC lesions and adjacent unaffected colon tissues (independent sample of 5 patients). Histone H3 was used as a loading control. **C**. Gating strategy for FACS-sorting of cancer cells (GFP^{+}), granulocytes ($CD11b^{+}Ly6G^{hi}$), other myeloid cells ($CD11b^{+}Ly6G^{lo}$) and the stromal cells (GFP^{-}, $CD11b^{-}$, $Ly6G^{-}$) from experimental hepatic metastases. **D**. Immunoblotting for PAD4 performed on cell lysates of indicated human and mouse cancer cell lines and tumor lysates of experimental murine hepatic metastases. GAPDH was used as a loading control. Recombinant human PAD4 was used as a positive control. P – patient, N – adjacent unaffected tissue, M – metastasis, T – primary CRC lesions tissue.

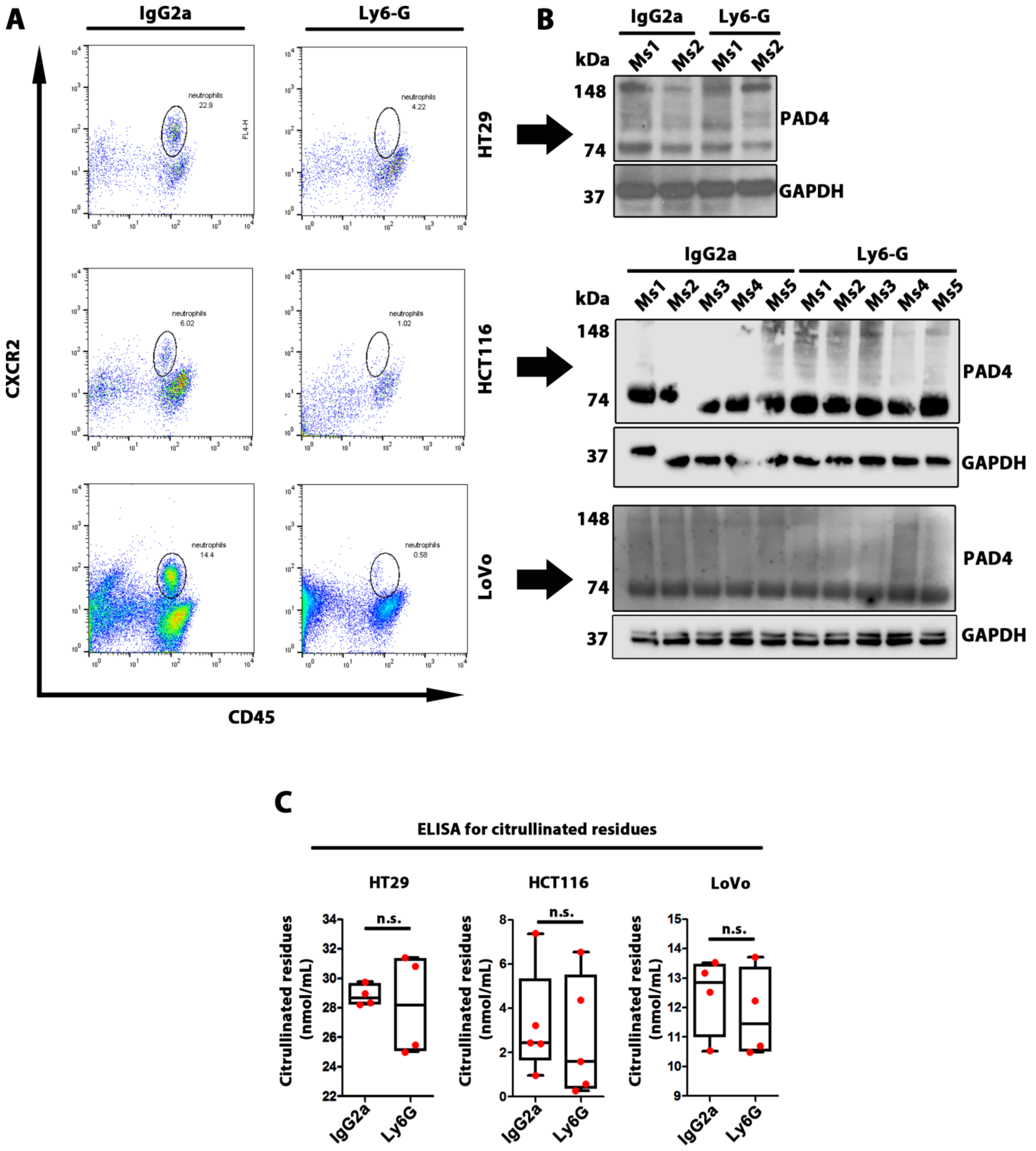

Supplementary Figure 7. Neutrophil depletion of experimental liver metastases does not alter intratumoral levels of PAD4 and/or citrullinated residues. **A**. Total hepatic CD45^{+}/CXCR2^{+} cell counts in metastasis-bearing mouse livers from animals treated with IgG2a or anti-Ly6G antibody (n=5 biological replicates per group). A representative flow cytometry profile is shown. **B**. Tumor lysates from metastasis-bearing livers from experiment in **A** were probed for PAD4 to examine the effect of neutrophil depletion on intratumoral PAD4 levels (n=5 biological replicates per group, except for HT29, n=2 replicates). Large arrows show corresponding data for each cell line in **A** and **B**. GAPDH was used as a loading control. **C**. ELISA for citrullinated residues in metastasis tissue lysates from experiment in **A** (n=5 biological replicates per group). Error bars indicate range, center values indicate median, box bounds indicate second and third quartiles (n.s. = not significant, Mann-Whitney U test). Ms – mouse, ELISA - the enzyme-linked immunosorbent assay.

A

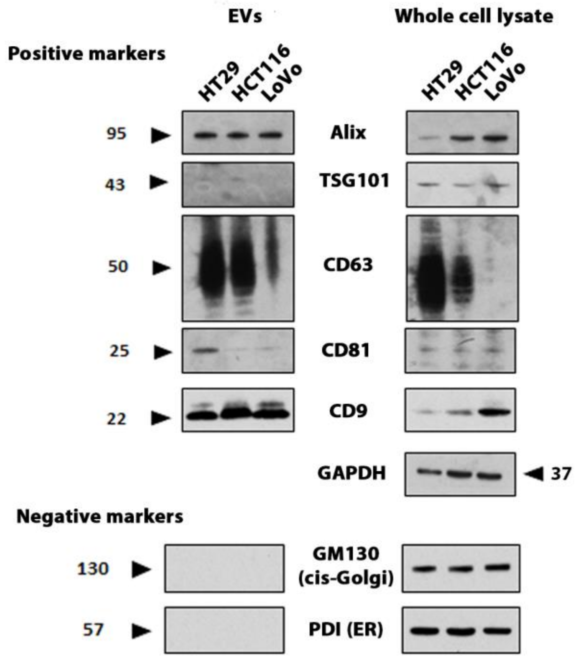

B

Cell line	Total PAD4 amount (ng, mean±SD)			Supernatant vs. EVs difference
	Conditioned media	Supernatant	EVs	
HT29	112.10±6.31	26.99±3.56	70.09±9.69	P = 0.0019
HCT116	38.77±2.63	14.87±1.72	24.88±4.52	P = 0.0231
LoVo	17.21±1.58	5.71±1.01	13.17±1.36	P = 0.0016

C

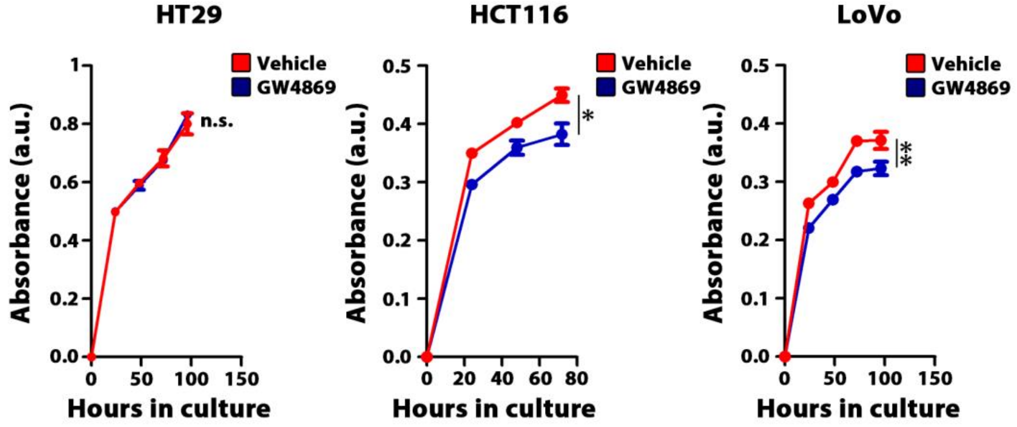

D

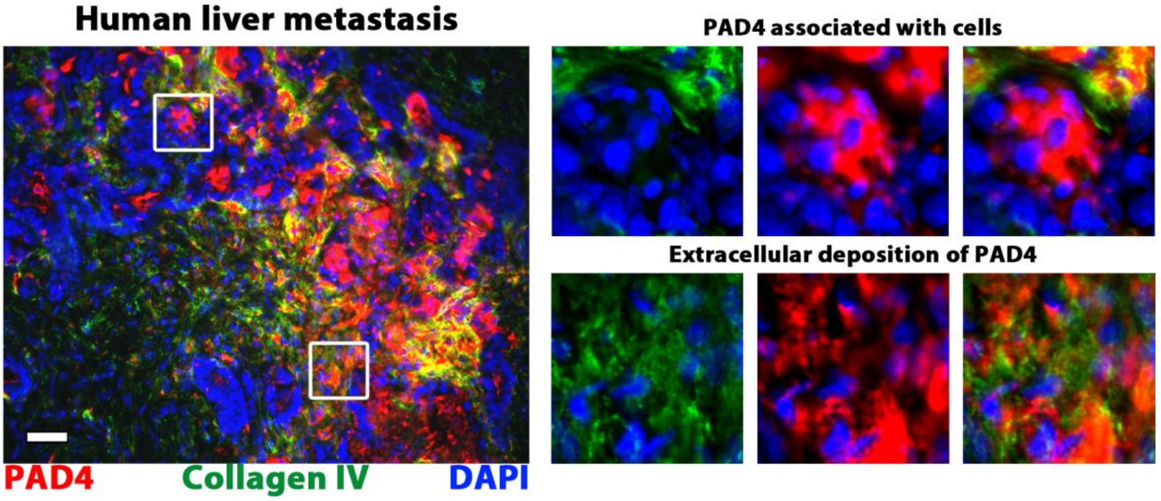

Supplementary Figure 8. Investigation of cancer-cell derived EVs and extracellular PAD4. **A**. Whole cell lysates (right) and EVs (left) were collected from cultured HT29, HCT116 and LoVo colon cancer cells and were probed for the indicated markers of extracellular vesicles (positive markers) and Golgi apparatus or endoplasmic reticulum markers (negative markers). GAPDH was used as a loading control. **B**. Serum-free conditioned media was collected from HT29, HCT116 and LoVo cells. EVs were pelleted by centrifugation and supernatant was collected. PAD4 was measured by ELISA in each compartment **C.** WST-1 proliferation and viability assay was performed in cultured HT29, HCT116 and LoVo colon cancer cells upon the addition of GW4869 (n=5 technical replicates per group). **D**. Representative image of immunostaining for PAD4 and collagen IV in human CRC hepatic metastasis tissue. Scale bar = 100 μm. For **C**, error bars indicate s.e.m. (*P < 0.05, **P < 0.01, n.s. = non-significant, two-way ANOVA). EV – extracellular vesicles, ER – endoplasmic reticulum, SD – standard deviation.

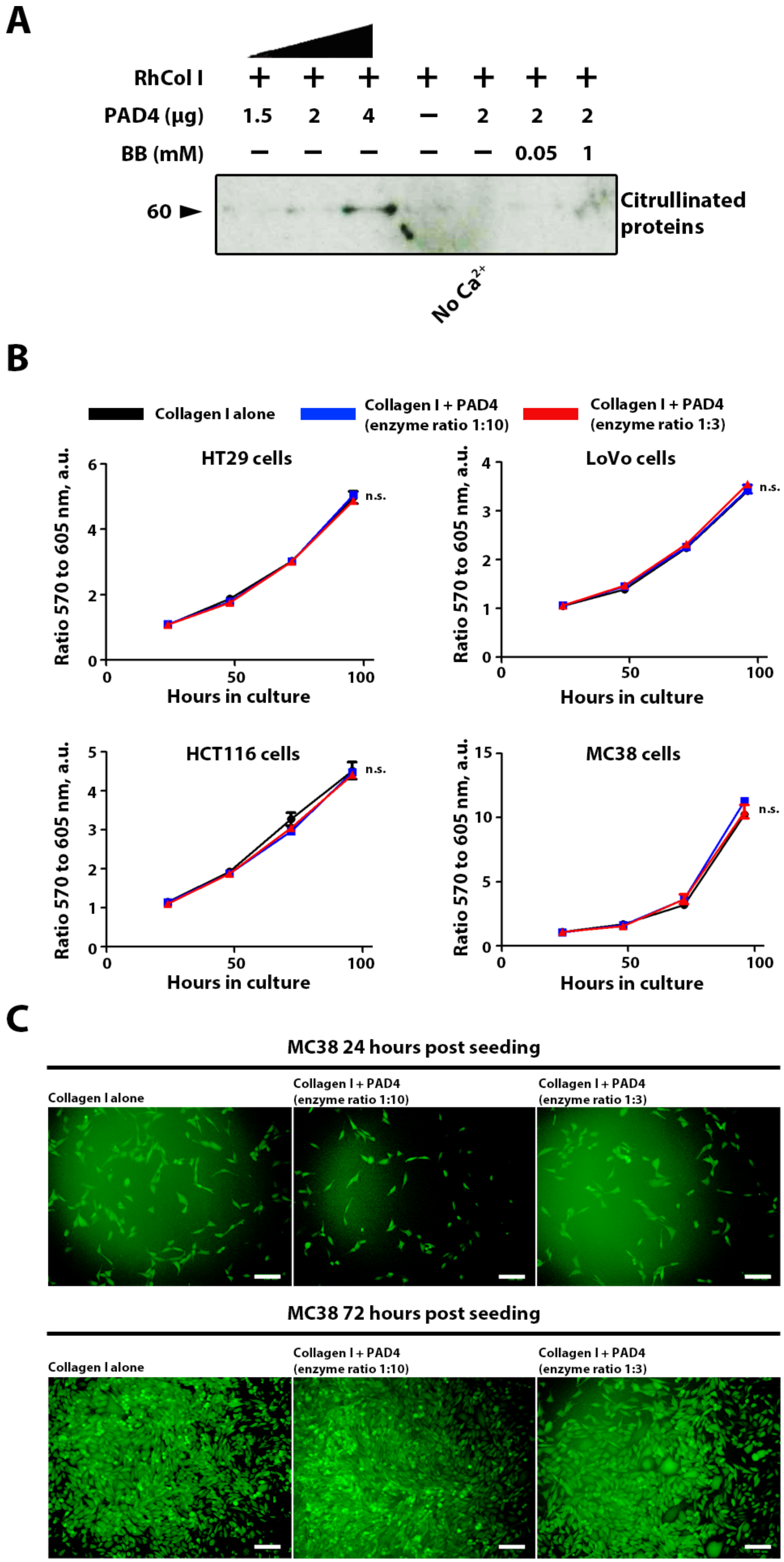
A
RhCol I + + + + + + +
PAD4 (µg) 1.5 2 4 – 2 2 2
BB (mM) – – – – – 0.05 1
60
Citrullinated proteins
No Ca2+
B
Collagen I alone
Collagen I + PAD4 (enzyme ratio 1:10)
Collagen I + PAD4 (enzyme ratio 1:3)
HT29 cells
LoVo cells
HCT116 cells
MC38 cells
Ratio 570 to 605 nm, a.u.
Hours in culture
n.s.
C
MC38 24 hours post seeding
MC38 72 hours post seeding
Collagen I alone
Collagen I + PAD4 (enzyme ratio 1:10)
Collagen I + PAD4 (enzyme ratio 1:3)

Supplementary Figure 9. Citrullination of collagen I does not affect proliferation or viability of CRC cells. **A**. Collagen type I pre-treated with recombinant PAD4 (ascending concentration) or collagen I pre-treated with recombinant PAD4 and BB-Cl-amidine (ascending concentration) were subjected to immunoblotting for citrullinated proteins. Negative controls were recombinant collagen type I alone or depletion of Ca^{2+} in the experimental group. Experiment was performed two times. B. Cancer cells were plated in wells pre-coated with either recombinant collagen I alone, or collagen I with added recombinant PAD4 with ascending enzyme-to-substrate ratios (1:10 and 1:3) (n=5-7 technical replicates per group). At time points 24, 48, 72 and 96 h cells were examined with Cell Cytotoxicity Assay Kit (ab112118). **C**. Representative images of GFP^{+} MC38 cells from **B**. Scale bar = 100 μm. Error bars indicate s.e.m. (n.s. = non-significant, two-way ANOVA). BB – BB-Cl-amidine.

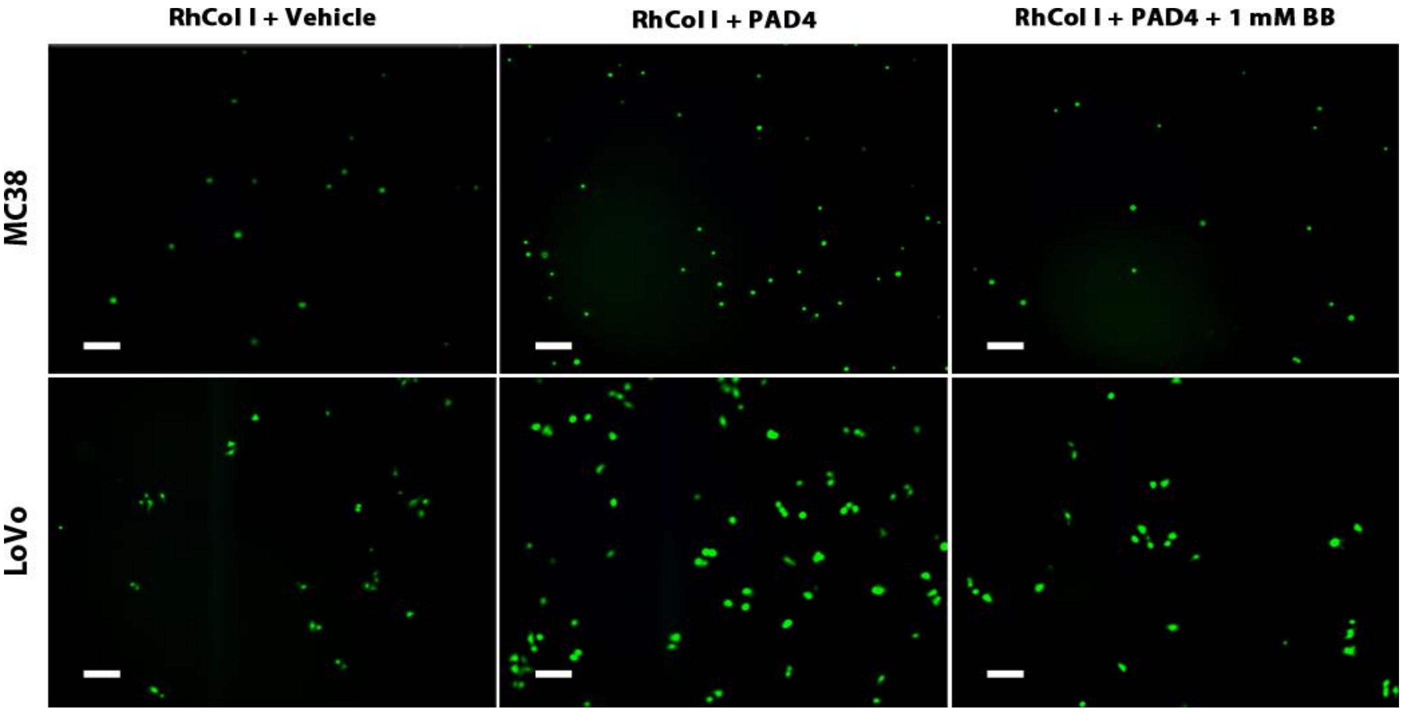

Supplementary Figure 10. Citrullination of collagen I alters adhesion of CRC cells. GFP+ cancer cells were plated in wells pre-coated with either recombinant collagen I alone, or collagen I with added recombinant PAD4, or collagen I with the addition of PAD4 and BB-Cl-amidine. Cells were imaged using an epifluorescence microscope. At least 5 fields of view per condition were taken. Shown are representative images of cells. Scale bar = 100 μm.

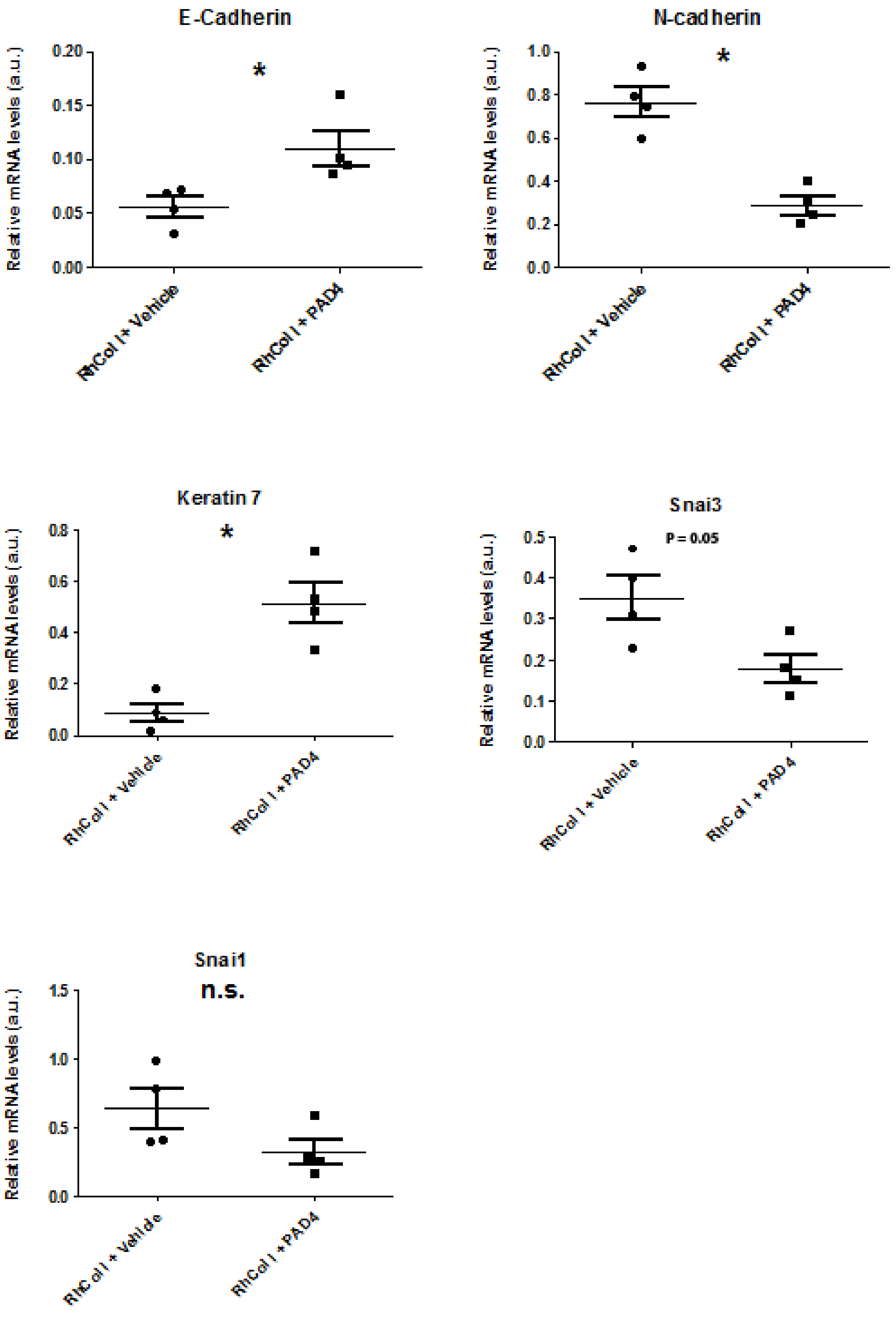
E-Cadherin
*
Relative mRNA levels (a.u.)
0.20
0.15
0.10
0.05
0.00
RhCol I + Vehicle
RhCol I + PAD4
N-cadherin
*
Relative mRNA levels (a.u.)
1.0
0.8
0.6
0.4
0.2
0.0
RhCol I + Vehicle
RhCol I + PAD4
Keratin 7
*
Relative mRNA levels (a.u.)
0.8
0.6
0.4
0.2
0.0
RhCol I + Vehicle
RhCol I + PAD4
Snai3
P = 0.05
Relative mRNA levels (a.u.)
0.5
0.4
0.3
0.2
0.1
0.0
RhCol I + Vehicle
RhCol I + PAD4
Snai1
n.s.
Relative mRNA levels (a.u.)
1.5
1.0
0.5
0.0
RhCol I + Vehicle
RhCol I + PAD4

Supplementary Figure 11. Relative (to HPRT) mRNA expression of selected EMT genes in LoVo cells seeded on collagen type I pre-treated with recombinant PAD4 as compared to those seeded on intact collagen type I (n=4 technical replicates per group). Throughout, $*P < 0.05$, n.s. = non-significant, Mann-Whitney U test.

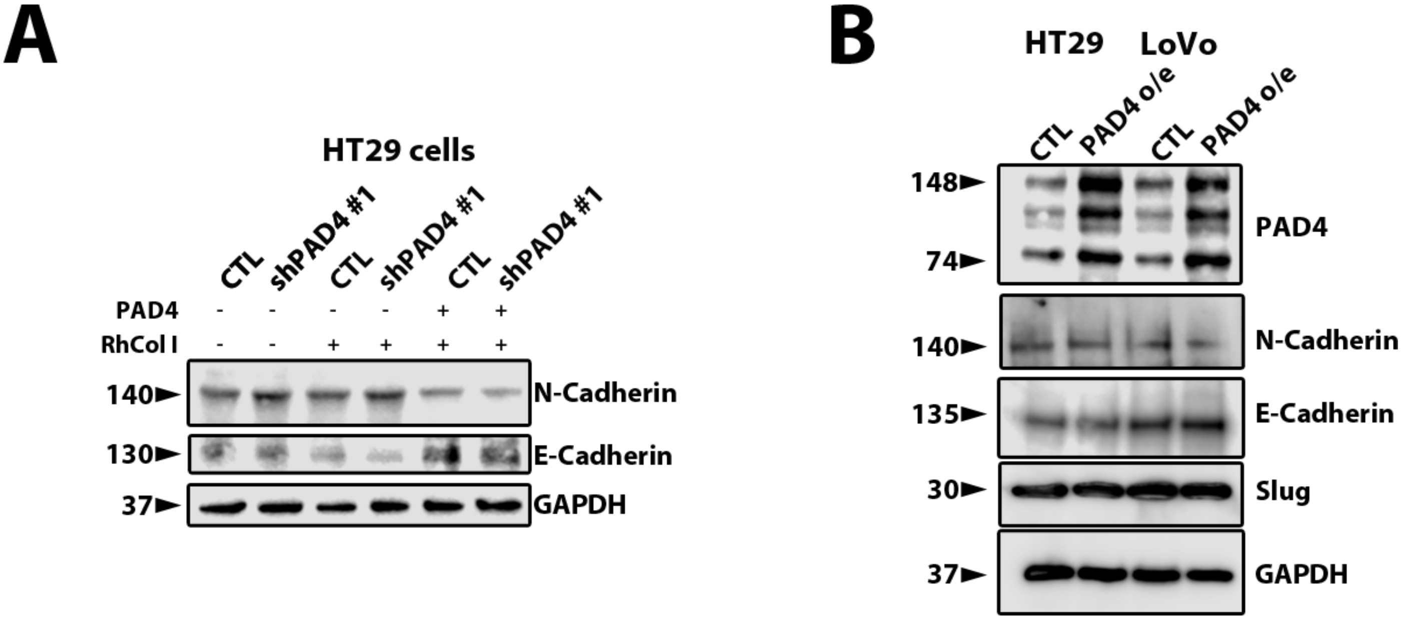

Supplementary Figure 12. Changes in epithelial-mesenchymal plasticity require extracellular but not intracellular citrullination. **A**. Control and PAD4-deficient HT29 cells were plated on recombinant collagen type I that had been previously incubated with recombinant PAD4 or vehicle as indicated. Cells were collected and probed for the indicated proteins. GAPDH was used as a loading control. **B**. Immunoblotting for indicated proteins in HT29 and LoVo cells transfected with empty vector (CTL) or PAD4-overexpressing lentivirus particles (PAD4 o/e). GAPDH was used as a loading control. CTL – control, shPAD4 – *PAD4* knockdown.

Fig3E

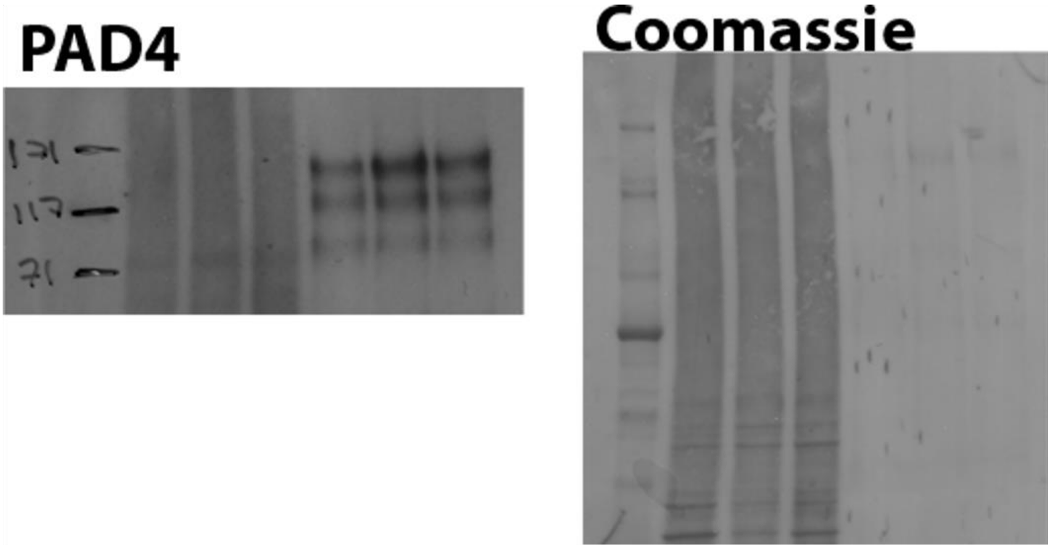

Supplementary Figure 13

Fig3G

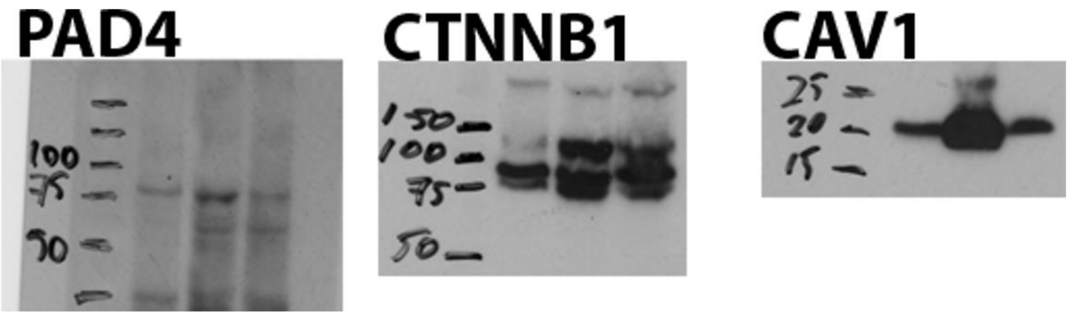

Supplementary Figure 13 (continued)

Fig3I

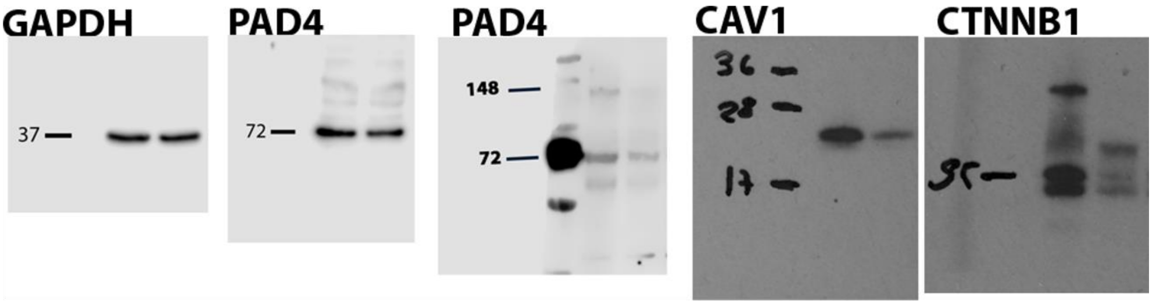

Supplementary Figure 13 (continued)

Fig4F

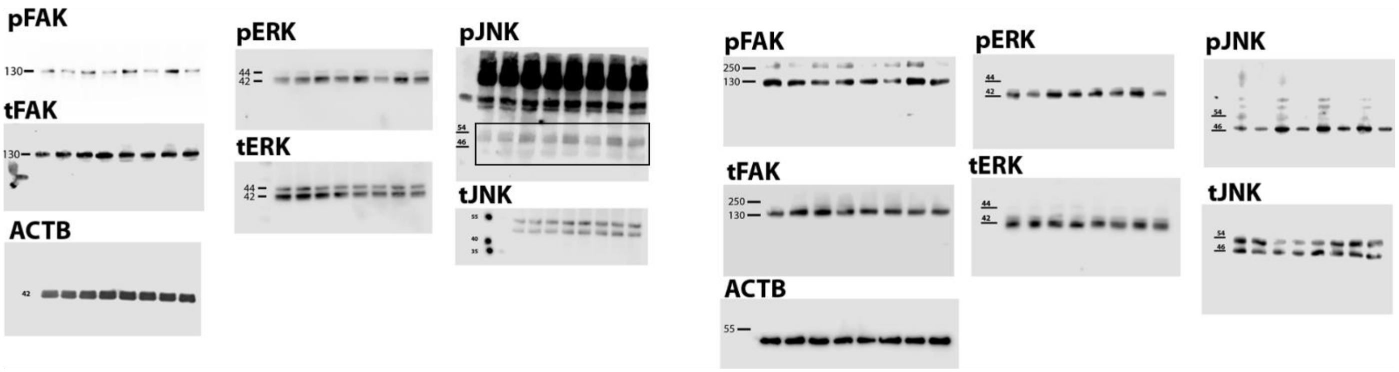

Supplementary Figure 13 (continued)

Fig4H

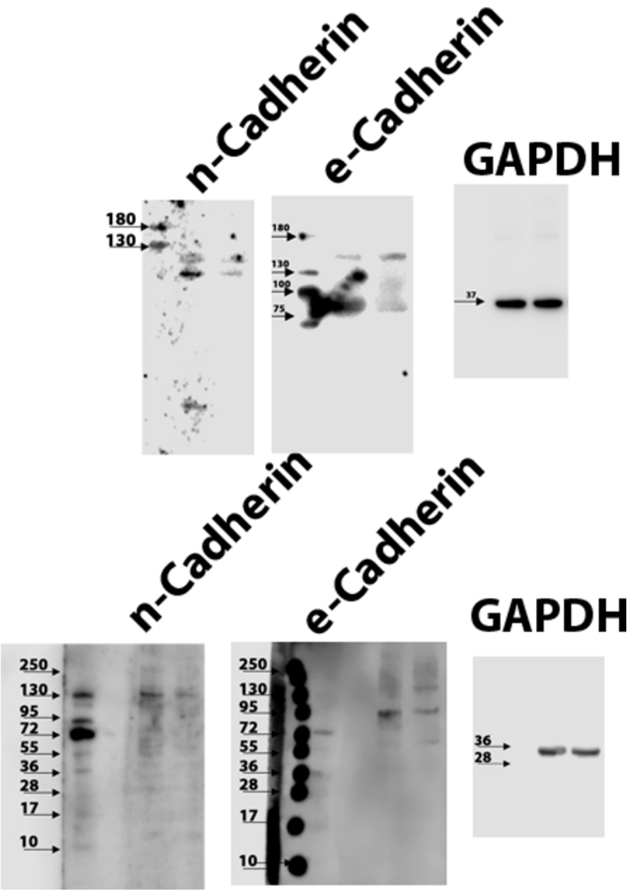

Supplementary Figure 13 (continued)

Fig5A,B

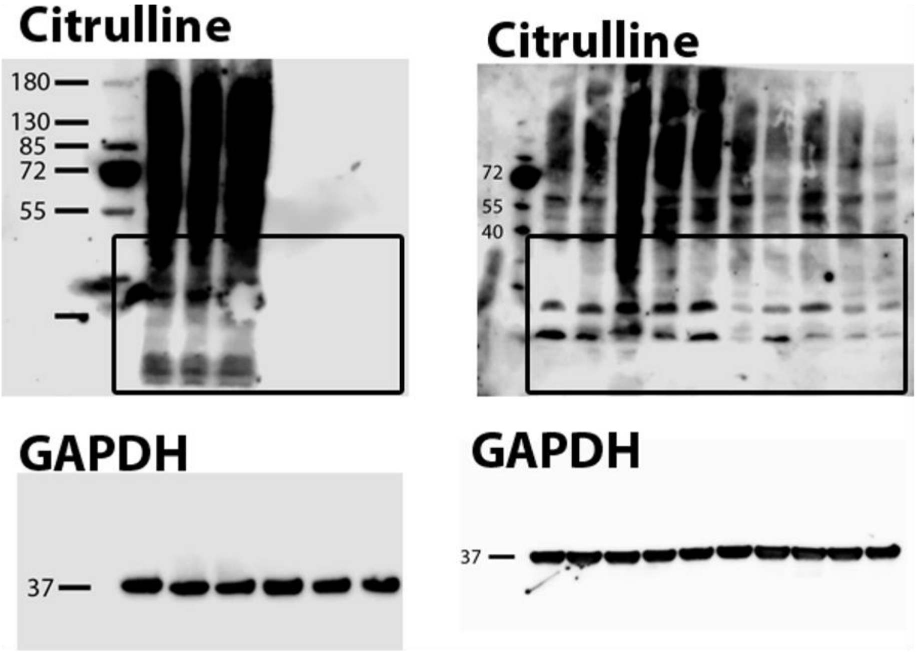

Supplementary Figure 13 (continued)

Fig6A

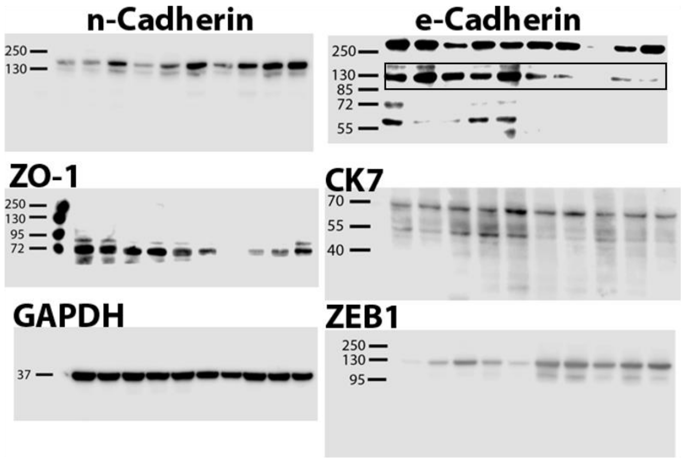

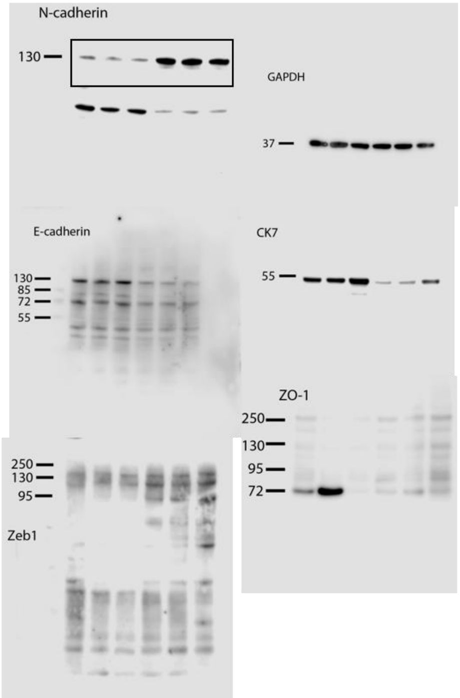

Supplementary Figure 13 (continued)

Fig6D

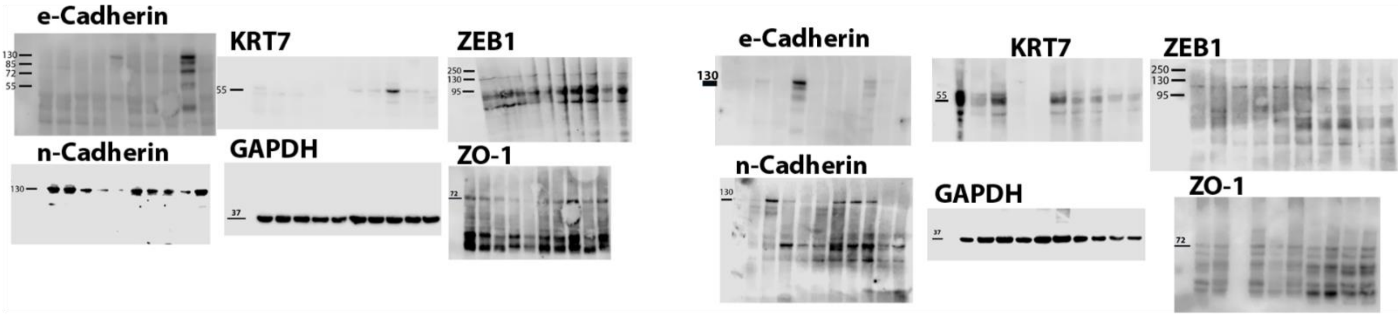

Supplementary Figure 13 (continued)

Fig7A,B

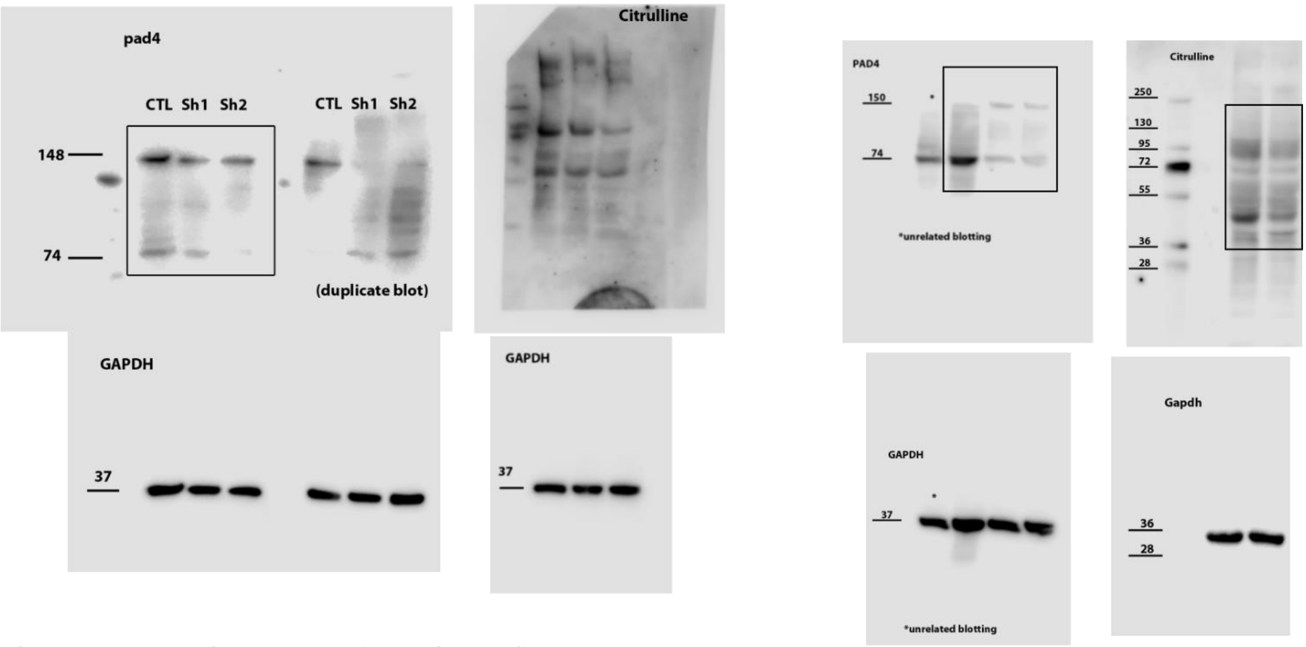

Supplementary Figure 13 (continued)

Fig7E

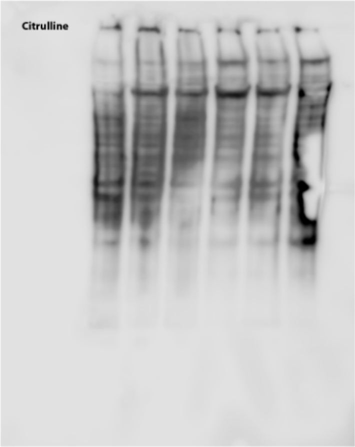

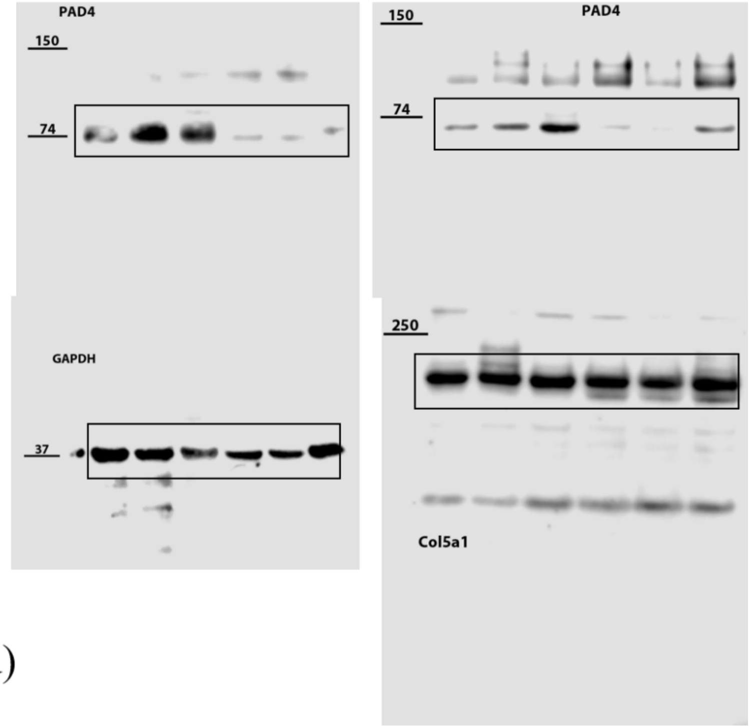

Supplementary Figure 13 (continued)

SFig4A

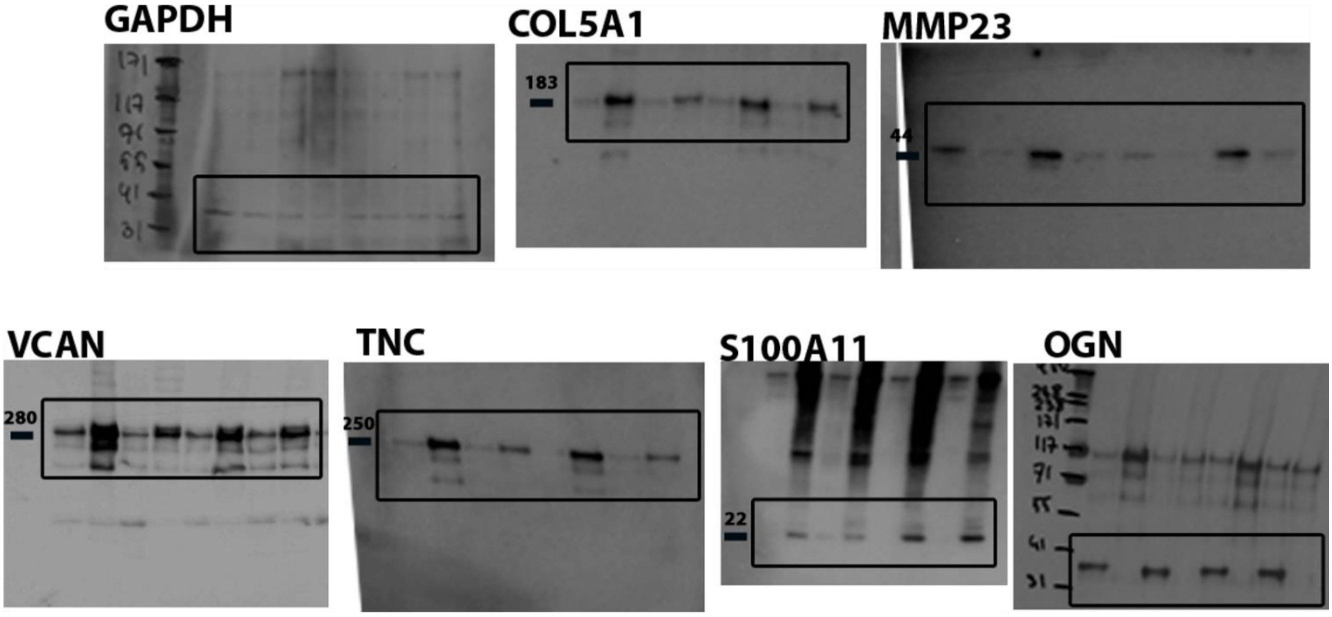

Supplementary Figure 13 (continued)

SFig6A,B

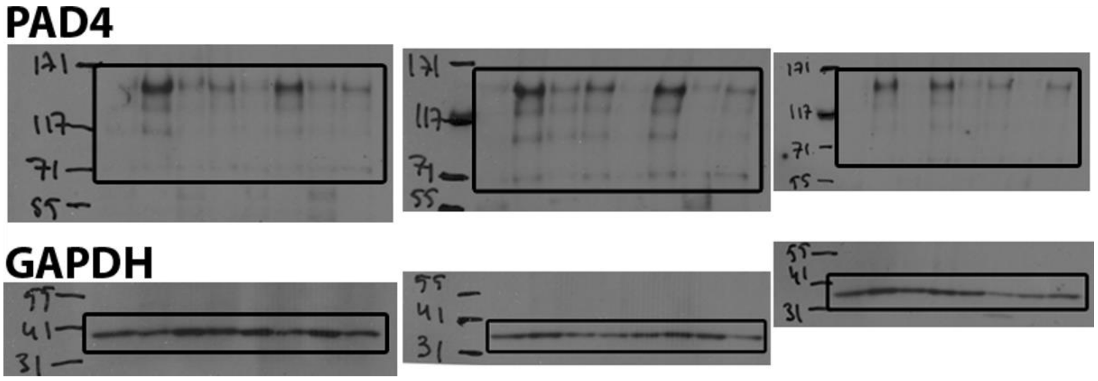

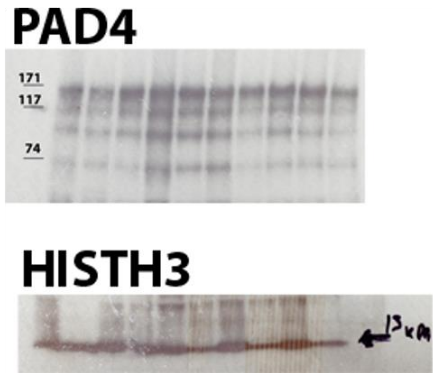

Supplementary Figure 13 (continued)

SFig6D

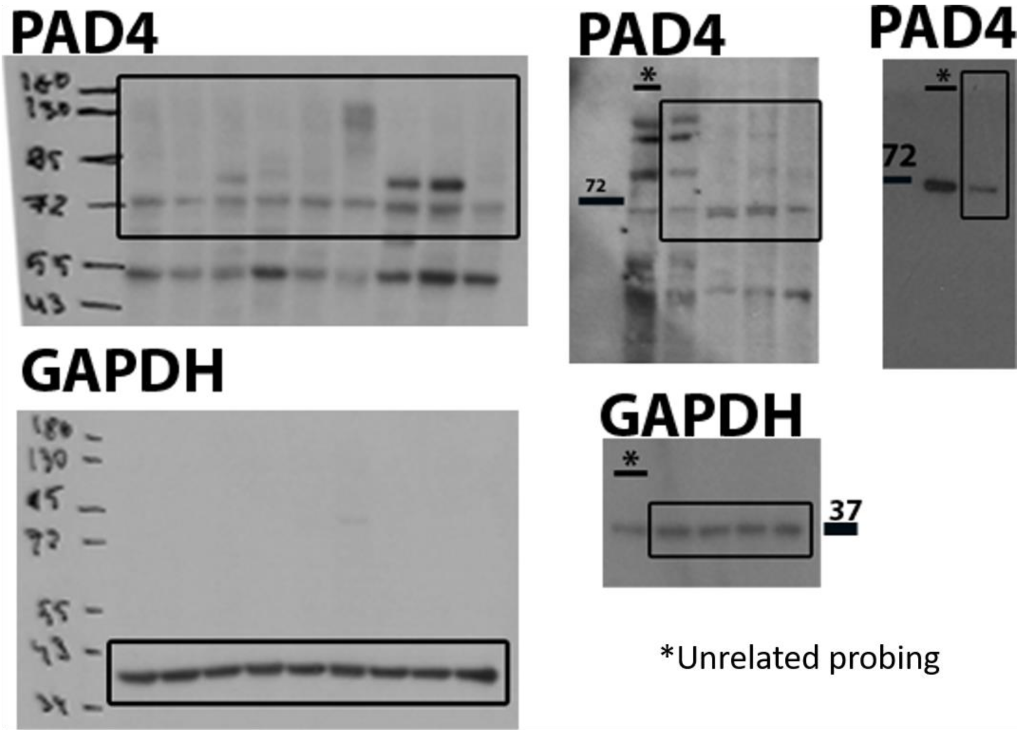

Supplementary Figure 13 (continued)

SFig7B

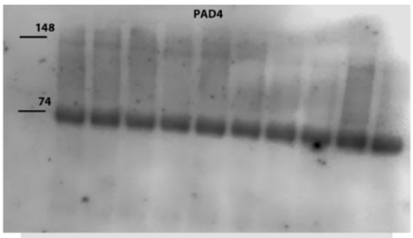

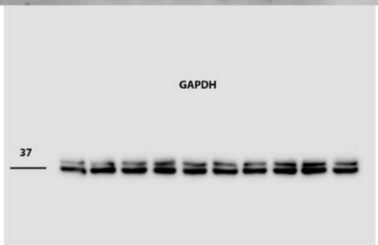

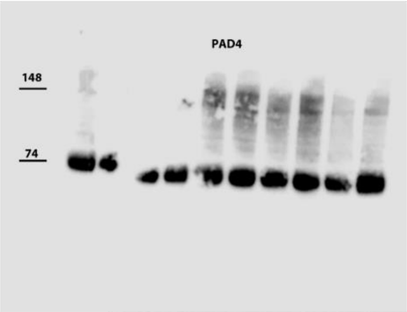

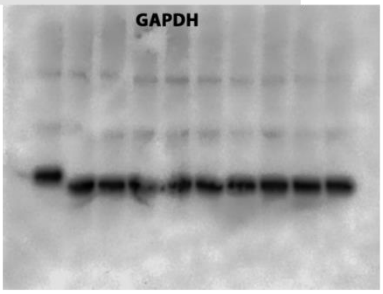

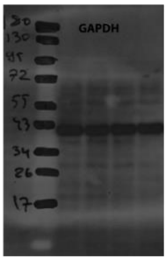

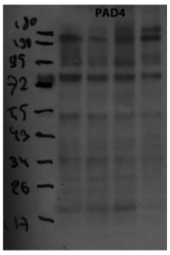

Supplementary Figure 13 (continued)

SFig8A (1)

Original WB

TSG101 WCL
PDI WCL & Exo
GM130 WCL & Exo
CD9 WCL & Exo
Taken from here

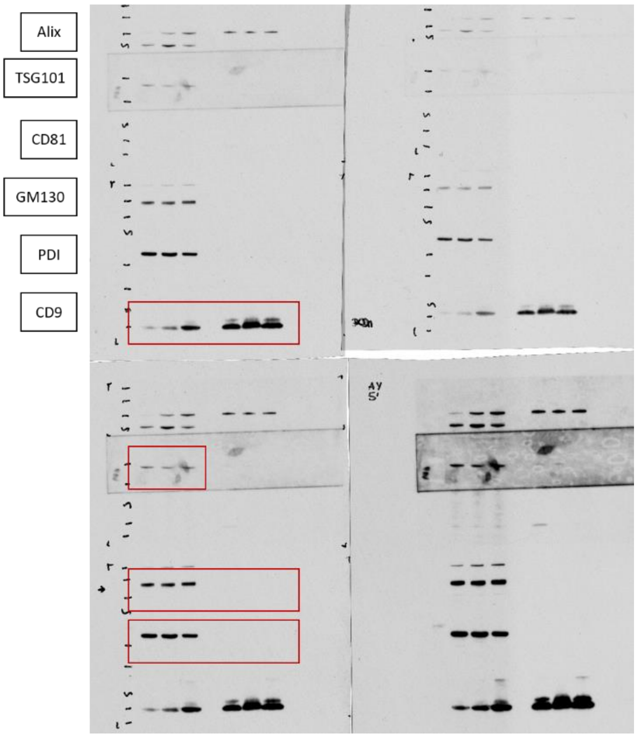

Supplementary Figure 13 (continued)

SFig8A (2)

Original WB

CD81 WCL & Exo
TSG101 Exo
Taken from here

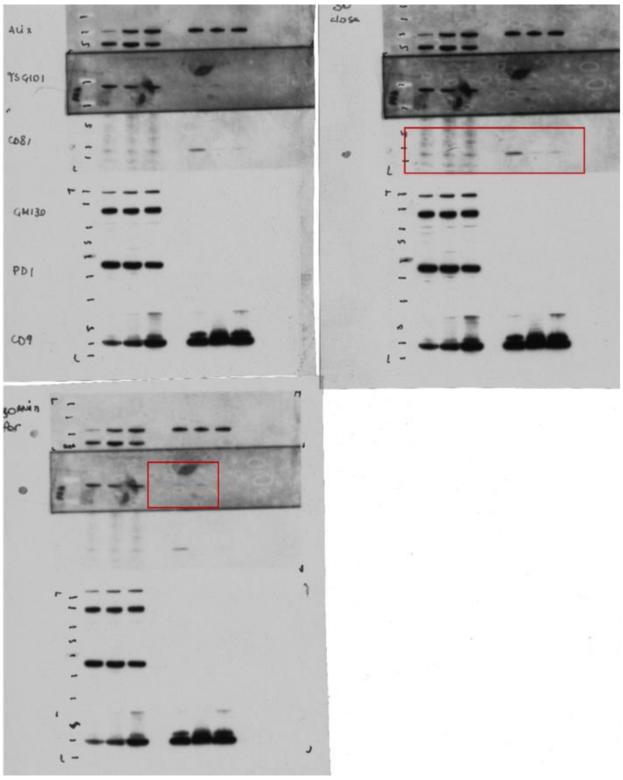

Supplementary Figure 13 (continued)

SFig8A (3)

Original WB

CD63 WCL & Exo
GAPDH WCL
Taken from here

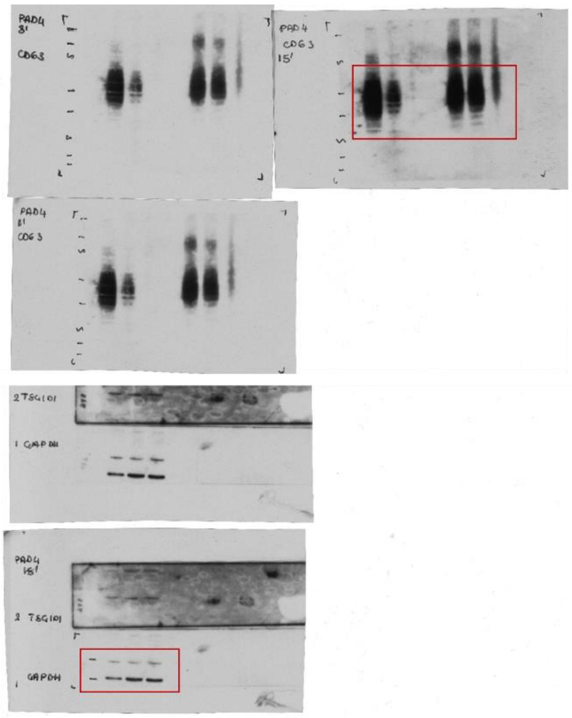

Supplementary Figure 13 (continued)

FigS10A

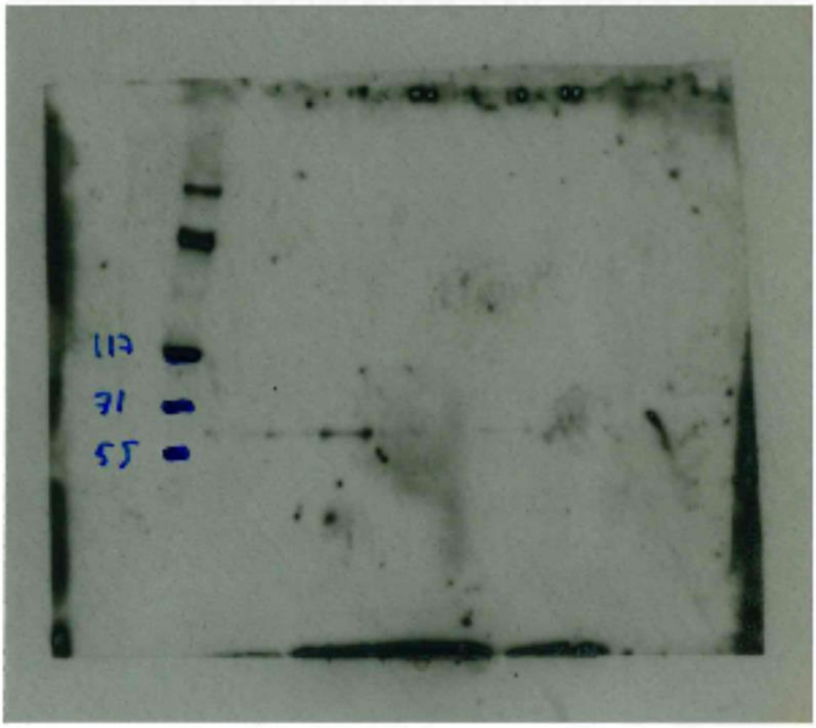

Supplementary Figure 13 (continued)

SFig11A

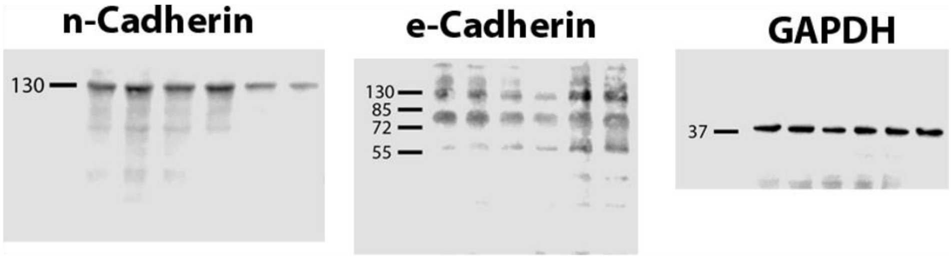

Supplementary Figure 13 (continued)

SFig11B

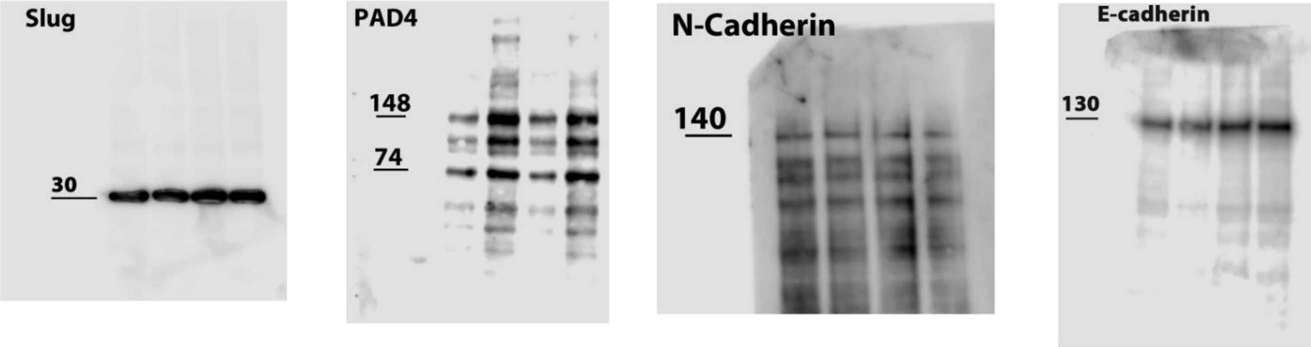

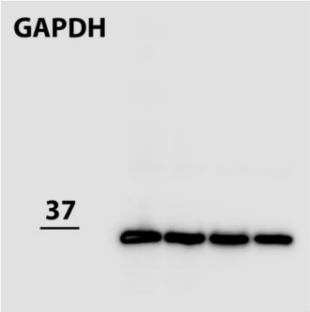

Supplementary Figure 13 (continued)
