# Supplementary material for: Colorectal cancer liver metastatic growth depends on PAD4-driven citrullination of the extracellular matrix
Source: Nat Commun. 2018 Nov 14;9:4783. doi: 10.1038/s41467-018-07306-7 (PMC6235861; doi:10.1038/s41467-018-07306-7)
Supplement: Supplementary file 3 — Description of Additional Supplementary Files [file 41467_2018_7306_MOESM3_ESM.pdf]

Description of Additional Supplementary Files

File Name: Supplementary Data 1
Description: Qualitative proteomics analysis of the matrisome of human liver metastasis

File Name: Supplementary Data 2
Description: Quantitative label-free proteomics analysis of the matrisome of human liver metastasis

File Name: Supplementary Data 3.
Description: Identification of the citrullinome of human liver metastasis ECM

File Name: Supplementary Data 4
Description: List of antibodies used in the study
